# Predicting future coexistence in a North American ant community

**DOI:** 10.1002/ece3.1048

**Published:** 2014-04-16

**Authors:** Sharon Bewick, Katharine L Stuble, Jean-Phillipe Lessard, Robert R Dunn, Frederick R Adler, Nathan J Sanders

**Affiliations:** 1 Department of Ecology and Evolutionary Biology, University of TennesseeKnoxville, Tennessee; 2 National Institute for Mathematical and Biological Synthesis, University of TennesseeKnoxville, Tennessee; 3 Center for Macroecology, Evolution and Climate, Natural History Museum of Denmark, University of CopenhagenCopenhagen, Denmark; 4 Quebec Centre for Biodiversity Science, Department of Biology, McGill UniversityMontreal, Quebec, Canada; 5 Department of Biological Sciences, North Carolina State UniversityRaleigh, North Carolina; 6 Department of Mathematics, University of UtahSalt Lake City, Utah; 7 Department of Biology, University of UtahSalt Lake City, Utah

**Keywords:** Ant communities, climate change, differential equations, mechanistic models, species interactions

## Abstract

Global climate change will remodel ecological communities worldwide. However, as a consequence of biotic interactions, communities may respond to climate change in idiosyncratic ways. This makes predictive models that incorporate biotic interactions necessary. We show how such models can be constructed based on empirical studies in combination with predictions or assumptions regarding the abiotic consequences of climate change. Specifically, we consider a well-studied ant community in North America. First, we use historical data to parameterize a basic model for species coexistence. Using this model, we determine the importance of various factors, including thermal niches, food discovery rates, and food removal rates, to historical species coexistence. We then extend the model to predict how the community will restructure in response to several climate-related changes, such as increased temperature, shifts in species phenology, and altered resource availability. Interestingly, our mechanistic model suggests that increased temperature and shifts in species phenology can have contrasting effects. Nevertheless, for almost all scenarios considered, we find that the most subordinate ant species suffers most as a result of climate change. More generally, our analysis shows that community composition can respond to climate warming in nonintuitive ways. For example, in the context of a community, it is not necessarily the most heat-sensitive species that are most at risk. Our results demonstrate how models that account for niche partitioning and interspecific trade-offs among species can be used to predict the likely idiosyncratic responses of local communities to climate change.

## Introduction

For at least the past 50 years, ecologists have sought to elucidate the mechanisms promoting coexistence in local communities (Hutchinson 1959; Chesson 2000; Amarasekare et al. 2004; HilleRisLambers et al. 2012). A major challenge over the coming decades will be to predict coexistence in local communities confronted with ongoing global change, including climate warming (Rozdilsky et al. 2001; Araújo and Rahbek 2006). Quantitative mechanistic models of coexistence can aid in predicting both the susceptibility of populations and the structure of communities under novel conditions (Burkett et al. 2005; Gilman et al. 2010; Walther 2010). In the context of climate change, these models should account for the combined roles of both climate-dependent and climate-independent species traits in governing community composition. The challenge is to account for both the direct effects of climate on individual species as well as the indirect effects of climate on interactions among species.

In ant communities, seemingly similar species often coexist locally (Kaspari et al. 2000; Andersen 2008). As a consequence, ant communities have long been model systems for the study of local coexistence (Levins and Culver 1971). A number of processes have been suggested to account for coexistence (or co-occurrence, in the case of neutral processes) in local ant communities, including the dominance–discovery trade-off, the dominance–thermal tolerance trade-off, spatial partitioning, niche partitioning, habitat complexity, and demographic stochasticity (Davidson 1977; Torres 1984; Cerda et al. 1997; Bestelmeyer 2000; Retana and Cerdá 2000; Palmer 2003; Sarty et al. 2006; Sanders et al. 2007; Andersen 2008; Stuble et al. 2013a). While many of these mechanisms focus on competition for food (Fellers 1987; Davidson 1998; Sanders and Gordon 2003; Lach et al. 2010), others also incorporate the influence of temperature (Cerdá et al. 1998; Bestelmeyer 2000; Lessard et al. 2009; Stuble et al. 2013a). The dominance–thermal tolerance trade-off, for example, suggests that subordinate ant species coexist with more aggressive ant species because they forage more heavily at suboptimal temperatures. If temperature shapes ant community structure, and if temperature is changing as a result of ongoing climate warming, then climate warming will almost certainly perturb ant community composition. However, while it is often possible to predict that climate change will affect a community, it can be much more difficult to predict how the change will occur.

The goal of this paper is to show how historical studies can be leveraged into predictions of future community responses to climate change based on a mechanistic modeling approach. Specifically, we develop a mathematical framework to identify the role of temperature in promoting current species coexistence. We then use this model to predict how the community will respond to a variety of climate change scenarios. To do this, we construct a temperature-dependent, hybrid dynamical model (Fagan et al. 2014) describing interspecific competition for food resource patches and species population dynamics. We then apply our model to a previously studied (Lynch et al. 1980) empirical system composed of three ground-foraging ant species [*Aphaenogaster rudis* (Emery), *Nylanderia faisonensis* (Trager), and *Prenolepis imparis* (Say)] that coexist in temperate forest communities throughout Eastern North America (Lessard et al. 2007, 2009; Stuble et al. 2013b). Using our model, we quantify the importance of temperature-dependent mechanisms for structuring the community. We then predict the effects of a variety of warming scenarios on the community and identify the species most sensitive to climate change. Although we present an analysis of one specific ant community (technically a “guild” sensu (Root 2001)) consisting of three species (Lynch et al. 1980), our broader goal is to show how mechanistic community-level models that incorporate temperature-dependent traits (e.g., foraging intensity) and temperature-independent traits (e.g., body mass) can explain contemporary community composition and predict community-level responses to future warming scenarios. This takes a first step toward addressing the recent call for more detailed models with more than two species and more realistic parameterization (Gilman et al. 2010). Using our model, we ask: (1) How important were different species traits (food discovery rate, food clearance rate, body mass, dominance hierarchy, and thermal niche) in determining community composition in 1979? (2) How will climate change affect community composition? (3) How does interspecific competition mediate shifts in community composition in response to climate change?

## Methods

### Model description

Our model measures ant performance in terms of resource collection (but see (Gordon 2013)) and thus tracks both resource patch dynamics and species abundance (Adler et al. 2007). We incorporate the environment by assuming that foraging efforts depend, in a species-specific manner, on temperature and thus, by proxy, season. Let *L* indicate 1 year in units of time, *t*. During year *y* (i.e., for *yL* ≤ *t* < *yL* + *L*), we can describe food patch dynamics according to


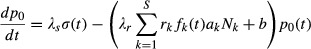
(1)


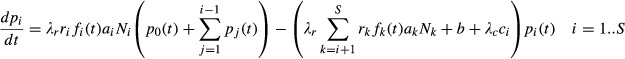
(2)


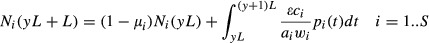
(3)

Here *p*_*i*_ is the number of discrete food patches per unit area that are occupied and defended by species *i*, *N*_*i*_ is the number of colonies of species *i* per unit area, *a*_*i*_ is the number of foraging ants per colony of species *i* (thus *a*_*i*_*N*_*i*_ is the total number of foraging ants of species *i* per unit area), and *p*_0_ is the number of unoccupied food patches per unit area. The model assumes a total of *S* species, numbered according to increasing behavioral dominance. *λ*_*r*_*r*_*i*_ is the per capita discovery rate of food patches by species *i*, *w*_*i*_ is the individual mass of species *i*, *μ*_*i*_ is the colony death rate of species *i,* and *λ*_*c*_*c*_*i*_ is the rate of patch removal by ant species *i*. Notice that both discovery rates and clearance rates are divided into two components – the first component (*λ*_*r*_ or *λ*_*c*_) is a scale factor that is constant across all species and that contains all relevant units. The second component (*r*_*i*_ or *c*_*i*_) is dimensionless and specifies rates relative to some standard (we use *N. faisonensis* rates as standards). In general, we will set *λ*_*r*_ = 1. This defines our units of time and area in terms of the *N. faisonensis* discovery rate (i.e., the unit of time is the time that it takes for one *N. faisonensis* worker to discover a single food patch located in one unit area). In contrast, we will use *λ*_*c*_ as an indicator of the average size of food patches in a particular environment (large patches will be cleared slowly and thus will have small *λ*_*c*_ values; small patches will be cleared quickly and thus will have large *λ*_*c*_ values). The remaining parameters in the model also reflect resource characteristics. *b* is the rate of patch removal by nonfocal species. It is a measure of the level of foraging by nonfocal species in the area. *ε* describes conversion of food patches to new ant colonies. It is a measure of food quality (all else being equal, higher quality resources will give a larger number of new colonies per food patch). *λ*_s_ describes the seasonal maximum rate of food patch production. It is a measure of how many patches, regardless of size or quality, enter the system per unit area, per unit time. Both resource and ant phenology are defined based on normalized, annually periodic functions. *f*_*i*_(*t*) is the fraction of the maximal foraging effort that is exhibited by species *i* at time *t*. Similarly, *σ*(*t*) is the fraction of the maximum food production that occurs at time *t*.

A simplified solution to equations (1–3) can be found by assuming that patch dynamics are fast compared to the dynamics of seasonal changes in the environment. This assumption is, in general, reasonable, since the timescale for patch dynamics (i.e., the time that it takes for eq. 1–2 to reach equilibrium) is on the order of minutes to hours (see, for example, figure 10 in Lynch et al. (1980); all but one bait was discovered within 15 min, and bait occupancy was relatively stable and dominated by *P. imparis* after 2 h), whereas the timescale for seasonal changes in temperature is on the order of days to weeks. We thus find the following “quasi-steady-state” values for *p*_0_ and *p*_*i*_:


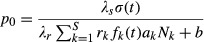
(4)


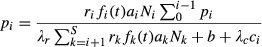
(5)

By substituting daily values for *σ* and *f*_*i*_ into equations (4) and (5), we can approximate solutions to equations (1–2) by solving sets of linear equations. This is done separately for each day of the year. Equation (3) can then be approximated by replacing the integral over the year with a sum over daily *p*_*i*_ values.

### System description

We apply our model to a well-studied ant community from the Smithsonian Environmental Research Center (SERC) in Anne Arundel County, Maryland, USA. (formerly the Chesapeake Bay Center for Environmental Studies). This community was studied in detail over 30 years ago (Lynch et al. 1980) and is an ideal test bed for our model for several reasons. First, the three most abundant ant species in this community, *P. imparis*, *N. faisonensis,* and *A. rudis*, are common in many deciduous forests in Eastern USA (Lynch et al. 1980; Kjar and Barrows 2004; Martelli et al. 2004; Lessard et al. 2007, 2009; Stuble et al. 2013a,b2013b). Thus, results from our model may be applicable to a variety of different forest ecosystems. Second, these same three species account for ∼70% of all species occurrences in the Maryland ant community (Lynch et al. 1980). Indeed, the next most common species, *Camponotus ferrugineus* (*Camponotus chromaiodes*), accounts for only 6% of total species occurrences, while the remaining ∼61 species (including *Aphaenogaster fulva*, *Myrmica punctiventris*, *Leptothorax curvispinosus* (*Temnothorax curvispinosus*), *Camponotus subbarbatus,* and *Formica pallidefulva*) each account for <3% of total species occurrences (Lynch et al. 1980). This creates a distinct division between abundant species and rare species, allowing us to reasonably approximate the responses of the abundant species to perturbations without considering the full diversity of ant species in the system (Adler et al. 2007). Third, Lynch monitored worker abundance in the community throughout an entire year (Lynch et al. 1980), which is necessary for constructing a predictive model incorporating temperature effects (Dunn et al. 2007). Fourth, the three most abundant ant species show strikingly different seasonal foraging characteristics, making it likely that thermal niche partitioning and/or trade-offs associated with thermal tolerance play a role in structuring the community. In particular, whereas both *A. rudis* and *N. faisonensis* foraging peaks in mid-summer, *P. imparis*, also known as the “winter ant”, forages most intensely in the fall and spring, with a drop in foraging over the summer (Talbot 1943b; Tschinkel 1987; Dunn et al. 2007). Finally, there is a clear and strict behavioral dominance hierarchy in the community (Lynch et al. 1980; Lessard et al. 2009; Stuble et al. 2013a), and many of the necessary rate parameters can be estimated either from the Lynch study itself (Lynch et al. 1980) or from experiments performed in other, related systems (Lynch 1981).

### Natural history

#### Prenolepis imparis

Ants of this species are opportunistic feeders (Fellers 1987), collecting a wide variety of insects, spiders, centipedes, and earthworms (Talbot 1943b; Fellers 1987). However, they will also collect plant material, for example fruit and plant exudates (Talbot 1957). Colonies of *P. imparis* can be either monogynous or polygynous, with a higher frequency of monogyny in northern locations (Tschinkel 1987). In favorable habitats, colony densities of 0.22 nests·m^−2^ have been observed, yielding ∼278 ants·m^−2^ (Talbot 1943a). Nests are dug into the soil (Talbot 1943a), and, at SERC, an average colony contains approximately 1200 ants (Lynch et al. 1980).

#### Aphaenogaster rudis

Like *P. imparis*, ants of the *rudis* group are general scavengers (Lubertazzi 2012). Much of their diet consists of small invertebrates or parts of insects; however, they will also collect mushroom species, elaiosome-bearing seeds, and liquids (Zelikova et al. 2008; Ness et al. 2009; Lubertazzi 2012). Colonies of *A. rudis* are functionally monogynous (Crozier 1973) and can occur at high densities of 0.5–1.3 nests·m^−2^ (Talbot 1957; Morales and Heithaus 1998; Lubertazzi 2012), yielding ∼ 430 ants·m^−2^ (Talbot 1957). Nests occur in the soil, in dead wood, under rocks, and in leaf litter (Lubertazzi 2012). At SERC, colonies contain approximately 300 ants (Lynch et al. 1980).

#### Nylanderia faisonensis

*Nylanderia faisonensis* appear to be dietary generalists (King 2007). They nest in rotten wood or shallowly in soil under leaf litter (Forster 2005; MacGown and Brown 2006; LaPolla et al. 2011) and can reach extremely high nest densities of 3.1 nests·m^−2^ (Lynch et al. 1988). At SERC, average colony size is approximately 125–150 ants (Lynch et al. 1980).

### Model parameterization

Below, we outline our basic approach for estimating each parameter in equations (1–3). A more complete description can be found in Appendix A1. Very generally, parameters in our model can be divided into three distinct categories: (1) constant parameters defining ant species attributes, (2) constant parameters defining resource attributes, and (3) seasonally varying parameters defining species or resource phenology.

#### Ant species attributes

Parameters describing species-specific ant characteristics are, for the most part, derived from Lynch et al. (1980). However, neither discovery rates, *r*, nor colony death rates, *μ*, were measured. To estimate discovery rates, we use our own data, previously collected in Great Smoky Mountains National Park (GSMNP), USA. Briefly, we use pitfall trap data, paired with baiting trials, and then estimate discovery rates using a maximum likelihood approach. A complete description of our empirical and estimation methods can be found in Appendix A1. For colony death rates, we use values reported in the literature (Tschinkel 1987; Wilson and Hölldobler 1990; Keller 1998). Thus, like discovery rates, estimates of colony death rates are representative, but not system specific.

#### Resource attributes

Three parameters define resource characteristics: *λ*_*c*_ determines the size of food patches, *λ*_*s*_ describes the rate of food patch production, and ε establishes food quality. One final parameter, *b*, reflects the rate of food loss due to nonfocal species. None of these parameters can be estimated from (Lynch et al. 1980). However, rather than arbitrarily selecting a single value for each of these parameters, we instead run replicate simulations over large numbers of randomly selected parameter sets. We then report our model predictions as summaries across simulation results. Each parameter is sampled over the range defined in Table 1, and, in all cases, parameters are sampled from a uniform distribution. Model results in Figs 2A, 3B, and 4B reflect the fraction of parameter sets (i.e., resource characteristics) within the parameter space that we sample (i.e., resource ranges defined in Table 1) that give rise to a particular community outcome. Model results in Figs 2B, 3C, and 4C reflect relative species abundances averaged across all sampled parameter sets (i.e., 
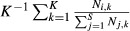
, where *N*_*i,k*_ is the number of colonies of species *i* for parameter set *k*, and *K* is the total number of parameter sets). For the sake of reference, we refer to different sets of resource parameters (*λ*_*s*_, *λ*_c_, *ε*, *b*) as defining specific “microhabitats”. We do this because different microhabitats should vary in terms of the types of resources and resource characteristics (i.e., resource parameters) present. Thus, at least from the perspective of a foraging ant community, we can fully characterize the relevant features of different microhabitats by specifying the differences in resource parameters between microhabitats.

**Table 1:** Model parameters used to simulate the empirical Maryland system under 1979 climate conditions. When exact parameter values are not available, and for all parameter values associated with microhabitat, reasonable ranges (see Appendix A1) are suggested. Species-specific parameters are reported in vector form, with all vectors ordered according to increasing behavioral dominance; thus, the first entry is always the value of the parameter for *Nylanderia faisonensis*, while the third entry is always the value of the parameter for *Prenolepis imparis*

Resource Characteristics and Species Traits	Parameter	Value/Range	Units	References
Dominance hierarchy (subordinate to dominant)	Implemented through model formulation	1. *N. faisonensis*	Dimensionless	Lynch et al. (1980)
		2. *A. rudis*		
		3. *P. imparis*		
Per capita discovery rate	*r* = [*r*_1_, *r*_2_, *r*_3_]	*r*_1_ = 1	Dimensionless	Empirical data see Appendix A1
		*r*_2_ = 2.2		
		*r*_3_ < 2.2		
Arbitrary scale factor^1^	*λ*_*r*_	1	Area·time^−1^·ant^−1^	
Worker mass	*w* = [*w*_1_, *w*_2_, *w*_3_]	*w*_1_ = 0.1	mg	Lynch et al. (1980)
		*w*_2_ = 1.3		
		*w*_3_ = 0.7		
Rate at which colonies clear patches	*c* = [*c*_1_, *c*_2_, *c*_3_]	*c*_1_ = 1	Dimensionless	Lynch et al. (1980)
		*c*_2_ = 6		
		*c*_3_ = 17		
Inverse patch size	*λ*_*c*_	0–200	Time^−1^	See Appendix A1
Max. rate of food patch production	*λ*_*s*_	0–200	Patches·time^−1^·area^−1^	See Appendix A1
Rate at which nonants clear patches	*b*	0.1–0.2	Time^−1^	See Appendix A1
Food quality	*ε*′ = *ε*/*μ*	0–2	Ants·mg·time·patches^−1^	See Appendix A1, Tschinkel (1987), Wilson and Hölldobler (1990), Keller (1998)

1 By setting *λ*_*r*_ = 1, we define our time and area units in terms of the discovery rate of *N. faisonensis*. Because we are only concerned with equilibrium solutions to equations (1–2), and because we are primarily interested in relative ant densities (i.e., populations per unit area), it is unnecessary to specify time and area units further.

#### Ant phenology

**Figure 1:**
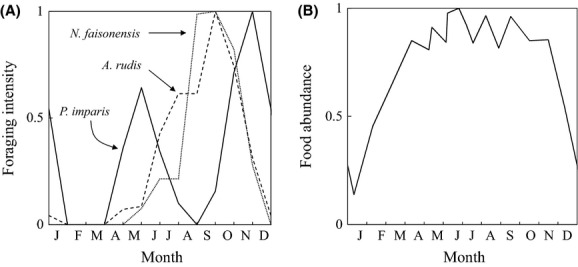
Ant and resource phenologies. (A) Seasonal foraging patterns for *Prenolepis imparis, Aphaenogaster rudis,* and *Nylanderia faisonensis*, reproduced from Lynch et al. (1980). (B) Seasonal abundance of understory arthropods (a proxy for food availability), reproduced from Lynch (1981).

Species-specific seasonal trends in foraging intensity are estimated from figure 1 in Lynch et al. (1980), reproduced in Fig. 1A. Very generally, foraging intensities for the first day of each month are taken directly from the figure and then linear interpolation is used to estimate foraging intensities on the days when no measurements were recorded. All foraging intensities are normalized so that the maximum value of *f*_*i*_ for each species is equal to one.

#### Resource phenology

Seasonal food availability was not measured in Lynch et al. (1980). However, in a separate study at a nearby SERC location, Lynch measured the overall seasonal abundance of understory arthropods (Lynch 1981). Description of the mature forest site in this second study (Lynch 1981) suggests that it is comparable to the SERC site in our study system; thus, we use these data to parameterize our model. Similar to foraging effort, food production on days when measurements were taken is read directly from figure 8 in Lynch (1981), reproduced in Fig. 1B. Linear interpolation is then used for days when no measurements were taken. As with foraging effort, seasonal food availability is normalized.

### Model analysis – historical description

With parameters based on Lynch et al. (1980), our model represents a historical description of the ant community at SERC. Using this description, we study the role of each species trait (food discovery rate, *r*, food clearance rate, *c*, body mass, *w*, dominance hierarchy, and thermal niche) in determining local coexistence. To do this, we set the trait of interest equal across all ant species, while leaving all other traits at their empirically parameterized values. To equalize food discovery and food clearance rates as well as body mass, we assign each ant species the same trait value, randomly selected from the range exhibited across all three species. To equalize behavioral dominance, we assume that each species has a 50% chance of winning/losing a food patch during a confrontation. Finally, to equalize thermal niche, we assume that all ant species forage all the time. We then run 10,000 simulations of equations (3–5). In addition to randomly selecting the equalized trait parameter for each simulation, we also randomly select parameters from the ranges specified in Table 1. Thus, each simulation represents slightly different resource characteristics and, by definition, comprises a different “microhabitat”. For each simulation, we record the species present at equilibrium, as well as their relative abundances. We then report the fraction of “microhabitats” with each species composition, as well as the relative abundances of each species averaged across all microhabitats.

### Model analysis – climate change

Next, we extend our analysis to consider the SERC ant community under future warming scenarios. To do this, we assume a number of likely perturbations to thermal niches. We then predict the effect that each perturbation would have on local coexistence and community composition. Specifically, we consider phenological shifts in foraging activity, temperature-dependent up- and downregulation of foraging intensity, and seasonal changes in food availability.

#### Phenological shifts

For many species, climate warming has been associated with earlier onset of spring activities and later onset (or offset, in the case of behaviors that are ceasing) of fall activities (Parmesan 2006). We characterize this type of phenological change by shifting ant foraging activity (Fig. 1A) and food availability (Fig. 1B) ahead by between 1 and 4 weeks for the months of March through July and behind by between 1 and 4 weeks for the months of September through January. Seasonal foraging patterns under 1–4 week phenological shifts are shown in Fig. 3A. The year-long pattern in food availability undergoes a similar transformation, which we do not show.

#### Temperature increases

Warming conditions are expected to up- and downregulate the foraging activities of *A. rudis*, *N. faisonensis,* and *P. imparis* in a species-specific manner (Lynch et al. 1980). To study how temperature-mediated regulation of foraging activity might affect community composition, we assume baseline temperatures according to monthly averages measured at SERC in 1984 (Correl et al. 1984) (the earliest year for which temperature data are available). We then consider uniform temperature increases of 1–5°C above baseline. This allows us to calculate seasonal foraging activity under warming conditions by scaling baseline foraging activity according to foraging temperature dependences measured in Lynch et al. (1980). For a more detailed description of how seasonal foraging patterns were calculated, see Appendix A1. Seasonal foraging patterns under 1–5°C warming scenarios are shown in Fig. 4A. For these scenarios, we do not consider changes in baseline resource availability (see Fig. 1B).

#### Food availability

Changes in food availability resulting from climate change will likely exhibit strong seasonal characteristics. In other words, while food availability may increase during 1 month, it may remain constant or decrease during another. To study the role of food availability on community composition, we generate a Latin Hypercube Sample (LHS) containing 100,000 values on each parameter with a range in Table 1 as well as on food supplementation of 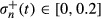 at the 17 measured time points across the year. The LHS grid is generated assuming a uniform distribution over all parameter and food supplementation ranges. Using the LHS grid, we then calculate partial rank correlation coefficients (PRCCs) for ant species abundances as a function of food supplementation at each of the 17 time points across the year. Positive and negative PRCCs indicate an increase and decrease in ant abundance, respectively, in response to food supplementation. Because our LHS grid samples over all microhabitat parameters, PRCCs calculated from our LHS sampling scheme reflect broad trends across microhabitats. Moreover, because the LHS grid allows for simultaneous changes in food supplementation across the season, reported trends for food supplementation at one time point are robust to variations in food supplementation at the other time points. Furthermore, because we supplement food at 17 discrete time points and then use linear interpolation for time points between these values, our model captures short-timescale correlations in climate-driven changes in food availability. However, such correlations are limited to timescales of ∼365/17 days.

### Model analysis – the role of interspecific competition

To determine the extent to which interspecific competition alters predictions regarding the effects of climate change, we run two separate simulations. In the first simulation, we run the full model in equations (3–5) and calculate percent change in abundance for each ant species according to the formula


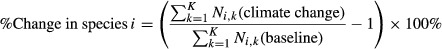
(6)

where, as above, *N*_*i,k*_ is number of colonies of species *i* for parameter set *k*, and *K* is the total number of parameter sets. In the second simulation, we run the model in equations (3–5) for each ant species separately. To do this, we set *N*_*j,k*_ = 0 for all *j* ≠ *i*, so that species *i* is the only ant present in the system. We then run the model and calculate the percent change in the abundance of ant species *i* according to equation (6). By comparing simulations with all three ant species present to simulations with only one ant species, we can determine the effects of interspecific competition on species responses to climate change scenarios.

## Results

### How important were different species traits in determining community composition in 1979?

**Figure 2:**
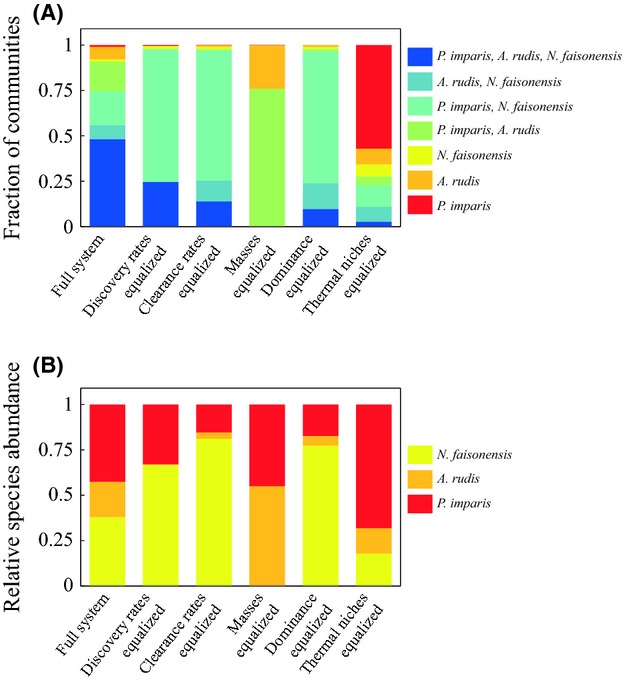
Community composition in the fully parameterized system as compared to systems where each different species trait is equalized. (A) Fraction of microhabitats with each possible species combination. (B) Abundance of each species averaged across all microhabitats.

As expected, the fraction of microhabitats in which all three species coexist is largest in the fully parameterized model where all traits differ across all species (Fig. 2A). This is also the scenario with the most even distribution of species abundances (Fig. 2B) (Pielou's evenness index of 0.95 as compared to 0.77 for thermal niche, 0.63 for body mass, 0.61 for discovery rate, 0.59 for dominance, and 0.54 for clearance rate). Equalizing discovery rates, food clearance rates, or dominance has a negative effect on the relative abundances of *A. rudis* (decreases of 98%, 82%, and 73%, respectively) and *P. imparis* (decreases of 23%, 64% and 59%, respectively), and a positive effect on the relative abundance of *N. faisonensis* (increases of 76%, 114%, and 104%, respectively). In contrast, equalizing worker mass has a negative effect on the relative abundance of *N. faisonensis* (a decrease of 100%) and a positive effect on the relative abundances of *A. rudis* (an increase of 182%) and *P. imparis* (an increase of 6%).

Equalizing thermal niches has a complex effect on community composition. In this case, the relative abundance of *P. imparis* benefits most (an increase of 60% vs. a decrease of 28% for *A. rudis,* and a decrease of 53% for *N. faisonensis*). As compared to other traits, however, loss of thermal niche differentiation has the least severe effect on the evenness of the species distribution (Fig. 2B) (Pielou's evenness index of 0.77), but the most severe effect on local coexistence (Fig. 2A). Communities with a single ant species comprise over 72% of simulated microhabitats when thermal niches are equalized: three times greater than any other scenario. Similarly, when thermal niches are equalized, only 2.8% of simulated microhabitats contain all three ant species. This is lower than all but the scenario with equalized masses, which gives no simulated microhabitats with local coexistence of all three species. Overall, these results suggest that removing thermal niches yields increased microhabitat partitioning – all three species continue to persist; however, they no longer coexist within the same “microhabitat” (i.e., within a single simulation with a single set of resource characteristics). Instead, each ant species persists by competitively excluding the other species from a subset of microhabitats (i.e., one ant species dominates for each sets of resource characteristics). In other words, coexistence is no longer possible at the local scale (within one microhabitat), but can only occur over broader scales where multiple different microhabitats are present.

### How will climate change affect community composition?

#### Phenological shifts

**Figure 3:**
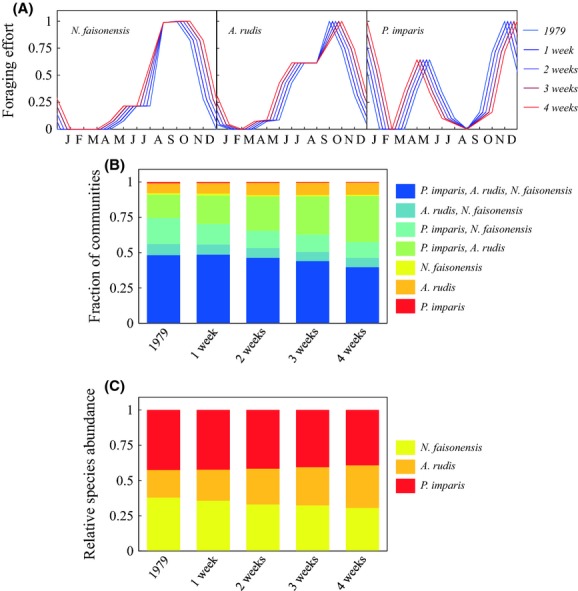
(A) Seasonal foraging activities of the three ant species from SERC under spring and fall phenological shifts of 1–4 weeks (i.e., extended summers of 2–8 weeks). (B) Fraction of microhabitats with each possible species combination for each different phenological shift. (C) Abundance of each species averaged across all microhabitats for each different phenological shift.

As expected, phenological shifts that extend the summer period (Fig. 3A) have a negative effect on the relative abundance of the winter ant, *P. imparis* (a maximum decrease of 8%, Fig. 3C). Surprisingly, however, phenological shifts that extend summer have an even stronger negative effect on the relative abundance of *N. faisonensis* (a maximum decrease of 19%, Fig. 3C), despite the fact that *N. faisonensis* is most active during the summer months, such that one might naively expect it to benefit from an increased summer period. In contrast to both *N. faisonensis* and *P. imparis*, the relative abundance of *A. rudis* responds positively to extended summer scenarios (a maximum increase of 55%, Fig. 3C). Interestingly, the fraction of microhabitats that support coexistence among all three ant species declines (a maximum decrease of 18%) under extended summer scenarios (Fig. 3B). Communities with three ant species are largely replaced by two species ant communities comprised of *A. rudis* and *P. imparis* (Fig. 3B).

#### Temperature increases

**Figure 4:**
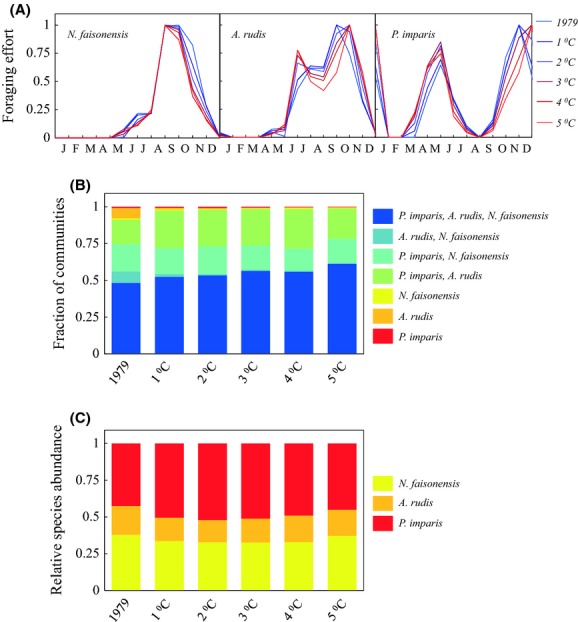
(A) Seasonal foraging activities of the three ant species from SERC under year-wide temperature increases of 1–5°C. (B) Fraction of microhabitats with each possible species combination for each different temperature increase. (C) Abundance of each species averaged across all microhabitats for each different temperature increase.

Year-round temperature increases of 1–5°C produce similar but distinctly different foraging patterns (Fig. 4A) as compared to pure phenological shifts (Fig. 3A). Under temperature increase scenarios, spring foraging by *P. imparis* shifts to earlier dates and increases in intensity relative to fall foraging. Similarly, spring foraging by *A. rudis* shifts to earlier dates, fall foraging shifts to later dates, and summer foraging decreases – an effect that may be related to the recently documented heat susceptibility of *A. rudis* (Pelini et al. 2012). In contrast, *N. faisonensis* foraging fails to exhibit any phenological shifts, although there is an overall decrease in spring and fall foraging intensity.

Temperature increases have a negative effect on the relative abundance of *N. faisonensis* (a maximum decrease of 14%, Fig. 4C). This negative effect on abundance is most severe for intermediate temperature increases of 1–3°C. Temperature increases also have a negative effect on the relative abundance of *A. rudis* (a maximum decrease of 22%, Fig. 4C), a result not seen in the phenological scenarios. Again, this effect is most severe for intermediate temperature increases of 1–2°C. Surprisingly, increased temperatures have a positive effect on the relative abundance of *P. imparis* (a maximum increase of 22%, Fig. 4C). Even at temperature increases of 5°C, the relative abundance of *P. imparis* is larger (an increase of 6%) than it was under baseline conditions. Unexpectedly and in contrast to phenological results, the fraction of microhabitats that support coexistence among all three species actually increases with increasing temperature (a maximum increase of 27%, Fig. 4B).

The differing effects of phenology and temperature on local coexistence probably stem from the fact that *A. rudis* foraging is more sensitive to high temperatures than *N. faisonensis* foraging. In phenology change scenarios, *A. rudis* continues to forage heavily throughout the summer, putting extra pressure on the *N. faisonensis* population. This causes local extinction of *N. faisonensis* in microhabitats where the *N. faisonensis* population was already marginalized (notice that three species communities are largely replaced by *A. rudis* + *P. imparis* communities under phenology change, Fig. 3B). In contrast, higher temperatures actually lead to a reduction in *A. rudis* foraging during the hottest months of the year. Since *N. faisonensis* does not exhibit this same sensitivity, but rather continues to forage intensely throughout the summer, *N. faisonensis* is not as likely to be displaced and, in fact, may even begin to appear in some microhabitats where it was previously excluded by *A. rudis* (notice that communities with only *A. rudis* or *A. rudis* + *P. imparis* are replaced by three species communities under temperature change, Fig. 4B).

#### Food availability

**Figure 5:**
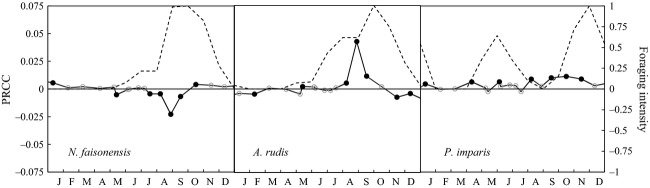
Partial rank correlation coefficients (PRCCs, solid line) for the impact of seasonal food availability on the abundances of *Nylanderia faisonensis*, *Aphaenogaster rudis,* and *Prenolepis imparis*. The figures include PRCCs significant at the 90% confidence level (filled black circles), PRCCs significant at lower confidence levels (open grey circles), and seasonal foraging intensities for each species (dotted line). Positive PRCCs suggest that food supplementation at a specific time point should increase the abundance of the ant species under consideration. Negative PRCCs suggest the opposite.

As expected, food supplementation during periods when an ant species is actively foraging tends to have a positive effect on the abundance of that species (Fig. 5). The correlation, however, is not perfect. For example, even though *N. faisonensis* forages intensely from July through October, increased food availability during this period of the year actually causes a decrease in *N. faisonensis* abundance (Fig. 5). In contrast, increased food availability in January can increase the *N. faisonensis* population, even though *N. faisonensis* does not forage intensely during this month.

### How does interspecific competition alter community response to climate change?

**Figure 6:**
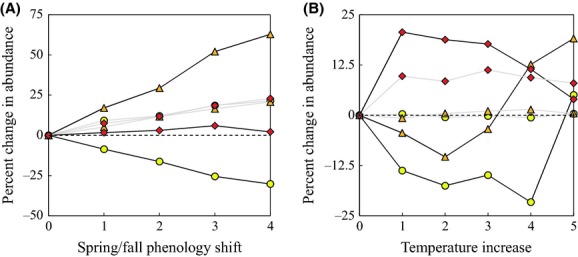
Percent change in the absolute abundance of *Nylanderia faisonensis* (yellow circles), *Aphaenogaster rudis* (orange triangles), and *Prenolepis imparis* (red diamonds) assuming (A) early spring/late fall phenological shifts of 1–4 weeks and (B) year-wide temperature increases of 1–5°C. Curves shown in grey assume that there is a single ant species present in the community (no interspecific competition). Curves shown in black assume that all three ant species are present in the community (interspecific competition).

When there is no interspecific competition, phenological shifts have a moderate positive effect on the absolute abundances of all three ant species (a maximum increase of 21% for *A. rudis* and *N. faisonensis,* and 23% for *P. imparis*, Fig. 6A). In contrast, in the context of interspecific competition, phenological shifts have a strong positive effect on the absolute abundance of *A. rudis* (a maximum increase of 63%), a strong negative effect on the absolute abundance of *N. faisonensis* (a maximum decrease of 30%), and very little effect on the absolute abundance of *P. imparis* (a maximum increase of 2%) (Fig. 6A). Similarly, when there is no interspecific competition, temperature increases have a moderate positive effect on the absolute abundance of *P. imparis* (a maximum increase of 11%) and very little effect on the absolute abundances of either *A. rudis* (a maximum increase of 1.4%) or *N. faisonensis* (a maximum decrease of 0.56%) (Fig. 6B). However, in the context of interspecific competition, moderate temperature increases of 1–2°C have a strong positive effect on the absolute abundance of *P. imparis* (a maximum increase of 19%), and negative effects on the absolute abundances of both *A. rudis* (a maximum decrease of 10%) and *N. faisonensis* (a maximum decrease of 18%). For larger temperature increases of 4–5°C, both the *A. rudis* and the *N. faisonensis* populations begin to recover, while the *P. imparis* population declines (Fig. 6B).

## Discussion

Ants have long been used as a prototypical system for the study of local coexistence and community effects (Brian 1956; Levins and Culver 1971; Levins et al. 1973; Davidson 1977). Recently, this work has been extended to include the examination of community responses to climate change (Pelini et al. 2011a,b2011b; Diamond et al. 2012; Warren and Chick 2013). In this paper, we consider mechanisms of coexistence in a common North American ant community, both under historical climate conditions from 1979 (baseline) and in the context of future warming scenarios. Analysis under baseline conditions (Fig. 2) suggests that several different factors contribute to local coexistence. For example, *P. imparis* is competitive (i.e., can collect sufficient food to persist) because it is the most aggressive ant species and also because it clears food patches fastest. In contrast, *A. rudis* is competitive because of its superior ability to discover food patches. Finally, *N. faisonensis* has the advantage of being small and thus producing a large number of workers per unit food. Models assuming no difference among species in a particular species trait always have a negative impact on the abundance of the species most superior for that trait and a positive impact on the abundance of the species most inferior for that trait.

These results agree broadly with conclusions from past studies that have implied a role for aggression/dominance (Fellers 1987; Cerda et al. 1997; Cerdá et al. 1998; Davidson 1998; Bestelmeyer 2000) and discovery rate (Davidson 1998; Adler et al. 2007; Lebrun and Feener 2007) in local coexistence among ant species. Unlike most trade-off studies (Davidson 1998; Holway 1999; Bestelmeyer 2000; Lebrun and Feener 2007), however, we consider multiple traits (and hence niche axes) that vary simultaneously across species (Lynch et al. 1980; Lynch 1981; Fellers 1989; Albrecht and Gotelli 2001). Because our model allows for niche partitioning in multiple dimensions, strict trade-offs are not necessary for coexistence. Moreover, trade-offs are not sufficient to explain coexistence in our system – a feature that we suspect is common, both across ant communities and across ecological communities more generally (e.g., (Stuble et al. 2013a)). The only traits that exhibit a strict trade-off in the Maryland ant community are discovery rate vs. body size, and months spent foraging (a crude proxy for thermal tolerance) vs. body size. Even when these trade-offs remain intact, significant reductions in coexistence can occur if there is loss of variation in other niche dimensions (Fig. 2). This highlights the difference between a trade-off contributing to species coexistence and a trade-off being necessary for coexistence. Again, the distinction is probably relevant in non-ant systems as well.

Past studies have also identified temperature/thermal tolerance as playing a role in local coexistence among ant species (Cerda et al. 1997; Cerdá et al. 1998; Bestelmeyer 2000). In our system, the effects of varying thermal niches are complex. In general, equalizing thermal niches increases the relative abundance of *P. imparis* while decreasing the relative abundances of both *N. faisonensis* and *A. rudis*. This suggests that *P. imparis* is more restricted by its thermal niche than are either of the other two species – a result in line with the cool weather foraging of *P. imparis* (Talbot 1943b; Tschinkel 1987). Interestingly, compared to equalizing any other trait, equalizing thermal niches has a less severe effect on the evenness of the species distribution, but a more severe effect on local coexistence. In other words, a lack of differences in thermal niches among species leads to increased microhabitat specialization.

Because we find a role for thermal niches in governing species composition and local coexistence amongst *A. rudis*, *N. faisonensis,* and *P. imparis*, we hypothesize that climate change may cause significant perturbations to this community. While one might predict that the most thermally intolerant ant species (*P. imparis*) would suffer most from warming trends, we find that this is not always the case. In fact, the *P. imparis* population actually increases both in relative abundance (Figs 3 and 4) and in absolute abundance (Fig. 6) in a number of warming scenarios. Surprisingly, *N. faisonensis* suffers most under all scenarios: spring/fall phenological shifts, year-wide temperature increases, and food supplementation during the majority of the productive year. Obviously, other warming scenarios are possible and may have an opposite effect on the *N. faisonensis* population. However, since we consider probable climate change trends, and since all of these predict a negative outcome for *N. faisonensis*, we identify *N. faisonensis* as more susceptible to population decline and local extinction in the face of climate change.

In our system, food availability and interspecific competition influence species responses to climate change. Food availability is important because it ameliorates the effects of warming on *P. imparis*. In particular, because food availability is relatively high in early spring and late fall, warming trends that push *P. imparis* foraging earlier or later in the year have a minimal effect on overall *P. imparis* abundance. Interspecific competition is important because it dramatically alters food partitioning and thus the amount of food available to each species. For example, under phenological change scenarios and in the absence of interspecific competition, all species abundances increase by approximately the same percentage (Fig. 6). This is because extended summers lead to an increase in food availability and thus an increase in the sustainable ant population. In the context of interspecific competition, however, the same phenological changes result in a large decrease in the *N. faisonensis* population and an even larger increase in the *A. rudis* population. This is because extended summers increase the length of time when *A. rudis* and *N. faisonensis* are foraging without interference from *P. imparis*. While this alleviates competitive pressure on *A. rudis*, the resulting increase in the *A. rudis* population causes increased pressure on *N. faisonensis*.

Similar effects of interspecific competition are apparent under temperature increase scenarios. In the absence of interspecific competition, temperature increases of 1–2°C result in more intense spring and fall foraging by *P. imparis*. This leads to increased food availability for the species and a corresponding increase in the *P. imparis* population (Fig. 6). The effects on *A. rudis* and *N. faisonensis*, however, are minimal. For *A. rudis*, this is because the increase in spring/fall foraging is compensated for by a decrease in summer foraging. For *N. faisonensis*, this is because small temperature increases have a minimal effect on the foraging window. Results are different, however, in the context of interspecific competition. For moderate temperature increases of 1–2°C, the large increase in *P. imparis* abundance results in increased competitive pressure on both *A. rudis* and *N. faisonensis*, and both populations suffer as a result (Fig. 6). At higher temperature increases of 4–5°C, *P. imparis* foraging is again restricted, and this allows for recovery of the *A. rudis* and *N. faisonensis* populations. Notice that interspecific competition also explains the less intuitive results from the food supplementation scenarios (Fig. 5). In particular, *N. faisonensis* is negatively affected by food supplementation during summer months, despite the fact that it is actively foraging over this period, because added food is disproportionately harvested by *A. rudis*.

Our finding that community context is important for predicting species responses to warming trends is in keeping with a number of recent empirical studies (Barton and Schmitz 2009; Barton et al. 2009; Walther 2010; Harley 2011). For example, just as food availability governs the impact of warming on *P. imparis* in our system, host plants appear to influence range expansion responses in butterflies (Hellmann et al. 2008). Likewise, temperature-dependent strength and/or nature of competition (mediated by effects on seasonal foraging in our system) is an important determinant of community composition across a broad range of communities (Jiang and Morin 2004; Poloczanska et al. 2008). These studies, like our own, reiterate a long-standing concern amongst ecologists (Kareiva et al. 1993; Davis et al. 1998a,b1998b; Pearson and Dawson 2003; Araújo and Luoto 2007) regarding predictive climate change modeling of biotic systems. In particular, they stress the danger of ignoring species interactions when predicting future community composition and identifying species susceptible to population decline under novel conditions. We would not, for example, recognize *N. faisonensis* as a “susceptible” species in any of the analyses above were it not for the effects of interspecific competition.

While the importance of species interactions has been used to argue for “process-based” models (Thuiller 2007; Morin and Thuiller 2009; Gilman et al. 2010), the dominant perspective for predictive ecological modeling in the context of climate change still focuses on broadly defined climate envelopes and species distribution models (Austin and Van Niel 2011; Fitzpatrick et al. 2011; Banta et al. 2012; Gillingham et al. 2012). Neither of these approaches accounts for species interactions. In this paper, we show how mechanistic models that account for species interactions can be constructed based on careful empirical studies of biological communities.

In theory, the approach that we outline could be applied to any system, regardless of the number of interacting species. In practice, however, the effort to characterize all relevant natural history traits for all species in even moderately diverse systems would be a challenge. Thus, particularly for diverse systems, simplifying assumptions are necessary. In our model, for example, we consider only the most abundant species. This approximation relies on the fact that rare species should have a minimum effect on abundant populations as a consequence of their scarcity (Magurran 2007). The approximation performs well when the total population size of rare species is small relative to the populations of the focal species themselves (in our study, for example, rare ant species constitute <30% of species occurrences (Lynch et al. 1980)). Even in highly diverse, tropical systems, a handful of abundant species often dominates (Longino et al. 2002).

For systems in which rare species cannot be ignored, our general modeling approach can still be applied; however, a different simplification is necessary. One obvious solution is to consider a few focal species, but then to incorporate the key effects of all additional species through fixed parameters. In the present study, we demonstrated this using the parameter *b* to account for competition between the focal ant species and all other species in the system. This approach works well if the “generic species pool” is large and diverse, making it relatively robust to changes in the system (i.e., there is compensation among species populations). When the effects of the generic species pool depend on context, our approach can still be used; however, such changes must be parameterized. In our case, for example, we could measure how food removal by nonfocal populations changes with climate or the abundances of the focal populations.

Even with the simplifications described above, the greatest obstacle to developing mechanistic models is still the added complexity associated with accounting for detailed natural history and species interactions. Ideally, models would include all aspects of natural history pertinent to predicting relevant species responses, but nothing more. However, it is often difficult to determine, a priori, which species traits and environmental features are important. Here, we suggest relying on natural historians who have years of insight and hard-earned data about their systems. In general, we advocate starting with the simplest model that can be agreed upon by modelers and field biologists alike. Added detail can be examined at a later date; however, if one begins with an overly complex model, it is often impossible to tease out predictions with any degree of generality. In our model, for example, we have not considered the role of colony structure on intraspecific competition, nor have we examined among-colony variation or any spatial aspects of the foraging process (Gordon and Kulig 1996). Furthermore, we have ignored daily trends in foraging intensity (Albrecht and Gotelli 2001; Stuble et al. 2013a). In addition, we have assumed that the success and/or failure of a species depends on how much food the species collects over an entire year. While this might be true for older colonies, the fate of younger, incipient colonies could very well depend on the food collected over months or even just days (Gordon and Kulig 1996). Finally, we have assumed that temperature, rather than photoperiod, is the dominant driver of colony phenology. While this is a reasonable assumption for many ant species (Kipyatkov 1993), factors regulating annual cycles for the specific species in our study have not been determined. Whether our conclusions will be robust to the inclusion of such additional detail remains an open question, although the generality of our results across a broad range of microhabitats and climate change scenarios makes this likely.

The best way to test the predictive capacity of any model is through new empirical characterization. The SERC ant community provides a particularly good system for this purpose because the original study was performed in 1979. Since then, significant climate change has occurred (McMahon et al. 2010; Hamburg et al. 2013). A test of the applicability of our model would thus be to return to the woodlot from the Lynch study and to repeat a full characterization of the ant community. Provided that there have been no other changes to the system (e.g., altered land use or introduction of invasive species), it should be possible to measure current seasonal food availability and ant foraging behavior and then re-parameterize our model for the climate conditions present today. This should result in new predictions for relative species abundances, which could be compared to the current composition of the ant community. For example, does our model accurately predict which relative species abundances have increased and which have decreased over the past 32 years?

The Lynch system is unique in being one of the few empirical systems in which comparisons of a broad number of system parameters can be made across a greater than 30 years timespan. Generally, though, we do not feel that a mechanistic community modeling approach to climate change requires such long-term experiments. Rather, we view mechanistic community models, particularly those that incorporate temperature-dependent parameters (e.g., foraging intensity in our model), as useful tools that can allow for prediction of long-term community composition based on short-term measurements of proximate responses to climate perturbation. Specifically, we suggest that models like the one presented here can extend short-term climate manipulation experiments to long-term community response predictions. Indeed, mechanistic models offer an invaluable approach for incorporating species interactions into predictive climate change models when experiments examining changes in species traits and species interactions under climate manipulation are available. As such, mechanistic community models with temperature-dependent parameters are likely to become an important tool for researchers trying to understand the complicated and often interdependent nature of biological community responses to climate perturbations.

## Supporting Information

**Appendix A1.** Model parameterization.

**Appendix A2.** Additional simulations.
