# Supplementary material for: Predicting future coexistence in a North American ant community
Source: Ecol Evol. 2014 Apr 16;4(10):1804–19. doi: 10.1002/ece3.1048 (PMC4063477; doi:10.1002/ece3.1048)
Supplement: Supplementary file 1 [file ece30004-1804-SD1.docx]

**Appendix A1 Model Parameterization**

Discovery Rates,$r_{i}$: Although the relative rates of movement of P. imparis, A. rudis, and N. faisonensis were discussed in the original study, per capita discovery rates for the three species were never determined systematically ([Lynch et al. 1980](#ENREF_31)). In the absence of this information, we use data from our own studies in Great Smoky Mountains National Park (GSMNP), U.S.A., to estimate relative discovery rates for A. rudis and N. faisonensis. We then suggest a reasonable range of discovery rates for P. imparis, which, at least during our baiting experiments, was not common enough in GSMNP to obtain a reliable discovery rate estimate. As described in ([Adler et al. 2007](#ENREF_13)), discovery rates are found using pitfall trap data paired with baiting trials (monitored at 15 minute intervals). Then, using the number of ants of species j in pitfall i, $n_{ij}$, the time to discovery of bait i by either species j or a more dominant species, $t_{ij}$, and the indicator function $f_{ij}$ where $f_{ij}=1$ ($f_{ij}=0$) if species j did (did not) find the bait before a more dominant species, we estimate the per capita discovery rate of species *j* by maximizing the likelihood

$L\left( r_{j} \right)=\prod_{j} \left( r_{j}n_{ij} \right)^{f_{ij}}e^{-r_{j}n_{ij}t_{ij}}$ (A.1)

assuming that bait trials are independent and that ants search for baits independently. This gives

$r_{j}=\frac{\sum_{i} f_{ij}}{\sum_{i} n_{ij}t_{ij}}$ (A.2)

Baiting was performed at ten different sites within GSMNP, and each site had between 10 and 12 independent bait/pitfall pairs, for a total of 115 baiting trials. N. faisonensis occurred in 17 of the pitfall traps and 63 of the baiting trials, whereas A. rudis occurred in 108 of the pitfall traps and 112 of the baiting trials. Our baiting experiments suggest that the discovery rate of A. rudis is approximately 2.2 times that of N. faisonensis. Since the discovery rate for P. imparis should be lower than that of A. rudis ([Lynch et al. 1980](#ENREF_31)) we consider a range of P. imparis discovery rates from $r\sim0$ to approximately twice the discovery rate of N. faisonensis. More generally, then, we define a vector of relative discovery rates, ordered according to increasing behavioral dominance (N. faisonensis first, A. rudis second and P. imparis third): $\boldsymbol{r}=\lambda_{r}\left[ 1, 2.2, r_{3} \right]$ where $\lambda_{r}$ is a scale factor and $r_{3}\in\left[ 0, 2.2 \right]$ is the discovery rate for P. imparis. In general, we will set $\lambda_{r}=1$, thereby defining our unit of time as the time it takes a single *N. faisonensis* worker to find a single patch of food located in a region exactly equal to one unit of area.

Clearance rates, c_i_, b : Clearance rates depend on the nature of the food source, with bigger food caches being removed proportionately slower. Nevertheless, we assume that relative rates of food clearance by the three species remain fairly constant, regardless of patch size. Experiments with juvenile crickets ([Lynch, Balinsky & Vail 1980](#_ENREF_3)) suggested that A. rudis and P. imparis clear food six and seventeen times faster than N. faisonensis, respectively. Although we acknowledge that the experiments with juvenile crickets did not control for discovery rate, behavioral dominance and abundance effects, we nevertheless use the results from these experiments to provide rough estimates for relative rates of resource removal. This gives $\boldsymbol{c}=\left[ 1, 6, 17 \right]$.

In the same set of experiments with juvenile crickets, it was noted that none of the 1240 crickets were removed by non-ants and that only 4.5% of the crickets were removed by non-focal ants ([Lynch, Balinsky & Vail 1980](#_ENREF_3)). This suggests that the focal ant species both discover and clear food patches faster than non-focal species, in other words $b\ll{\lambda_{r}\boldsymbol{r,}\lambda}_{c}\boldsymbol{c}$.

Ant masses, $w_{i}$: Mean dry weights (in milligrams) of twenty workers for each of the three ant species were measured ([Lynch, Balinsky & Vail 1980](#_ENREF_3)), allowing us to define a vector of ant masses, again ordered according to increasing behavioral dominance: $\boldsymbol{w}=\left[ 0.1, 1.3, 0.7 \right]$.

Colony death rates, $\mu_{i}$: The lifespans of both A. rudis and P. imparis colonies have been measured at ~ 9 years ([Tschinkel 1987](#_ENREF_4); [Wilson & Hölldobler 1990](#_ENREF_5); [Keller 1998](#_ENREF_2)). Although the lifespan of N. faisonensis colonies has not been determined, we suggest that it might be comparable to that of certain Lasius species, since they have similar life histories. Using the colony lifespan of L. alienus as a rough estimate for the colony lifespan of N. faisonensis, we assume that this species as well lives ~9 years ([Keller 1998](#_ENREF_2)). As a result, we assume that all ant species have similar colony death rates of $\mu_{1} = \mu_{2} = \mu_{3} = \mu\sim\frac{1}{9}yr^{-1}=2.1\times{10}^{-7}\mathrm{mi}n^{-1}$.

Colony size, a_i_: Although colony sizes for each of the three species are estimated ([Lynch, Balinsky & Vail 1980](#_ENREF_3)), we note that in the Lynch study, overall abundances are reported in terms of numbers of workers, rather than numbers of colonies. Defining worker number as W_i_ = a_i_N_i_ and substituting W_i_ into equations (1 - 3) we see that a_i_ drops out of the equations when they are reformulated in terms of worker abundance. Since we only consider worker abundance in the analysis that follows, the values of a_i_ do not influence model results.

Habitat variables: $\lambda_{c}$, $\lambda_{s}$, $\varepsilon$ and $b$ describe resource characteristics. $\lambda_{s}$ is the maximum rate of food patch production in the study region, $\lambda_{c}$ is the inverse size of the food patches in the study region, $\varepsilon$ (which we consider only as the compound parameter $\varepsilon^{'}=\frac{\varepsilon}{\mu}$) is the quality (i.e. energy/nutrient content) of the food patches in the study region and $b$ is the extent of foraging by non-ants. As suggested above, we expect $b\ll\lambda_{r}\boldsymbol{r,}\lambda_{c}\boldsymbol{c}$. Furthermore, since $\lambda_{r}=1$ is a rate constant associated with individual *N. faisonensis* foragers, while $\lambda_{c}$ involves recruitment of entire ant colonies, we expect $\lambda_{c}\gg\lambda_{r}=1$ (i.e. it takes ants longer to find food patches than it does to clear patches) except for extremely large food patches. Overall, this suggests that $\lambda_{c}\gg1\gg b$. However, this is as far as we can restrict resource characteristics based on ([Lynch, Balinsky & Vail 1980](#_ENREF_3)). This is not surprising, since $\lambda_{s}$, $\lambda_{c}$ and $\varepsilon$’ are likely highly variable depending on the specific types of resources found in different local regions. To accommodate this variability, we assume broad parameter ranges for each resource parameter. We then run replicate simulations of Eqs. (1-5) over large numbers of randomly selected parameter sets. We assume that each parameter set represents a unique microhabitat, and report model predictions as summary statistics over replicate samples (i.e. over microhabitats). To prove that our results are reasonably robust to the parameter ranges considered, we repeat our two climate change analyses with different parameter ranges (Appendix A2). While predictions are quantitatively different, most qualitative conclusions are the same for both parameter ranges.

Temperature-Dependent Foraging Patterns: Ant foraging patterns at SERC exhibit both a temperature-dependent component as well as an independent seasonal component ([Lynch, Balinsky & Vail 1980](#_ENREF_3)). To ensure that the seasonal component is maintained when estimating temperature responses, we compute foraging activity under warming conditions by scaling baseline foraging activity according to the fold-increase that would be expected given the foraging temperature-dependence. Specifically

$f_{i}^{W}\left( t \right)=\left\{ \begin{matrix} \frac{f_{i}\left( t \right)\varphi_{i}\left( T\left( t \right)+T_{w} \right)}{\varphi_{i}\left( T\left( t \right) \right)} & f_{i}\left( t \right),\varphi_{i}\left( T\left( t \right) \right)>0 \mathrm{or} \varphi_{i}\left( T\left( t \right)+T_{w} \right)=0 \\ \frac{f_{i}\left( t\pm1 \right)\varphi_{i}\left( T\left( t \right)+T_{w} \right)}{\varphi_{i}\left( T\left( t\pm1 \right) \right)} & f_{i}\left( t \right),\varphi_{i}\left( T\left( t \right) \right)=0 \mathrm{and} \varphi_{i}\left( T\left( t \right)+T_{w} \right)>0 \end{matrix} \right.$ (A.3)

where ‘+’ is used in the spring and ‘–’ is used in the fall. In equation (A.3), $T(t)$ is the baseline temperature at time t. $T_{w}$ is the temperature elevation (i.e. warming) above baseline. $f_{i}\left( t \right)$ is the fraction of the maximal foraging effort exhibited by species i at time t under baseline conditions (see above). $f_{i}^{W}\left( t \right)$ is the fraction of the maximal foraging effort exhibited by species i at time t under a $T_{w}$ temperature elevation. Finally, $\varphi_{i}\left( T \right)$ is foraging intensity as a function of temperature, T. $T(t)$ is obtained based on monthly average temperatures measured at SERC in 1984 ([Correl 1984](#_ENREF_1)). Specifically, we use the monthly average temperature on the first day of the month and then use linear extrapolation for all days in between. This allows us to generate a smooth curve for the yearly temperature profile. $\varphi_{i}\left( T \right)$ is obtained based on Figure 2 in [Lynch, Balinsky and Vail (1980](#_ENREF_3)), reproduced in Figure A.1. $\varphi_{i}\left( T \right)$ for measured temperature values are read directly from the figure, all other $\varphi_{i}\left( T \right)$ are determined by linear interpolation. The conditions on the r.h.s. of Eqs. (A.3) ensure that, when foraging intensity is zero under baseline conditions, change conditions can still result in non-zero foraging. This is accomplished by separately considering (1) months that give zero foraging under baseline conditions and non-zero foraging under change conditions versus (2) months that give zero foraging under both conditions, months that give non-zero foraging under both conditions or months that give non-zero foraging under baseline conditions and zero foraging under change conditions. For scenarios that fall into the first category, one month offsets are used to predict foraging under change conditions.


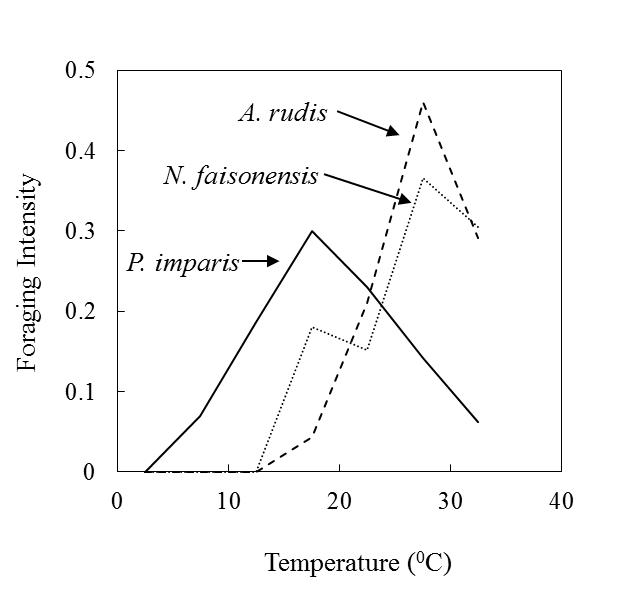


Figure A.1 Temperature-dependent foraging patterns for P. imparis, A. rudis and N. faisonensis, reproduced from ([Lynch, Balinsky & Vail 1980](#_ENREF_3))

**References for Appendix A1**

Correl, D., Jordan, T., Duls, J. (1984) Air temperature data from SERC weather station. SERC, SERC Weather Station, Edgewater, Maryland.

Keller, L. (1998) Queen lifespan and colony characteristics in ants and termites. *Insectes Sociaux,* **45,** 235-246.

Lynch, J.F., Balinsky, E.C. & Vail, S.G. (1980) Foraging patterns in three sympatric forest ant species, *Prenolepis imparis*, *Paratrechina melanderi* and *Aphaenogaster rudis* (Hymenoptera: Formicidae). *Ecological Entomology,* **5,** 353-371.

Tschinkel, W. (1987) Seasonal life history and nest architecture of a winter-active ant, *Prenolepis imparis*. *Insectes Sociaux,* **34,** 143-164.

Wilson, E.O. & Hölldobler, B. (1990) *The Ants,* 1 edn. Belknap Press of Harvard University, Boston.

**Appendix A2 Additional Simulations**

Figures A.2 and A.3 correspond to Figures 3 and 4 in the main text. However, we now consider restricted parameter ranges for the maximum rate of patch production, $\lambda_{s}\in\left( 0,100 \right)$, patch size, $\lambda_{c}\in\left( 0,100 \right)$ and patch quality $\varepsilon'\in\left( 0,100 \right)$. These ranges are half as large as the ranges used in the main text (see Table 1). We do not alter the range for $b$ since this parameter was already reasonably restricted based on empirical results ([Lynch, Balinsky & Vail 1980](#_ENREF_3)). Not unexpectedly, restricting the ranges of resource parameters to smaller values (i.e. a lower rate of food patch production, lower quality patches, larger patches) benefits *N. faisonensis* because it is a small species and can thus survive in more marginal habitat. Consequently, *N. faisonensis* abundance is, on average, higher (compare Figure A.2c with Figure 3c or Figure A.3c with Figure 4c). Likewise, a larger fraction of microhabitats (i.e. resource parameter sets) support communities with *N. faisonensis* present (compare Figure A.2b with Figure 3b or Figure A.3b with Figure 4b).

Nevertheless, trends with changing phenology or changing temperature are broadly similar for both the parameter ranges from the main text and the restricted parameter ranges. In particular, changing phenology generally results in an increase in the abundance of *A. rudis* at the expense of *N. faisonensis* (compare Figure A.2c and Figure 3c), and results in more microhabitats with *A. rudis* present and fewer microhabitats with *N. faisonensis* present (compare Figure A.2b and Figure 3b). Similarly, moderate increases in temperature generally result in an increase in *P. imparis* at the expense of both *A. rudis* and *N. faisonensis* (compare Figure A.3c and Figure 4c) and result in more microhabitats with *P. imparis* present and fewer microhabitats with *P. imparis* absent (compare Figure A.3b and Figure 4b); however, for both parameter ranges, this trend is reversed at large increases in temperature. Both parameter ranges also suggest that increased temperatures increase the number of microhabitats (i.e. resource parameter sets) that can support coexistence among all three species (compare Figure A.3b and Figure 4b). While changing phenology does the opposite when parameter ranges are large (Figure 3b), its effect on local coexistence of all three species is generally much smaller for the smaller parameter ranges (Figure A.2b). Thus, while large phenology mismatch still suggests a decrease in the number of microhabitats that can support coexistence of all three species, small phenology mismatches have no effect, or even increase the number of microhabitats with all three species present. This is the one difference between simulations over different resource parameter ranges.


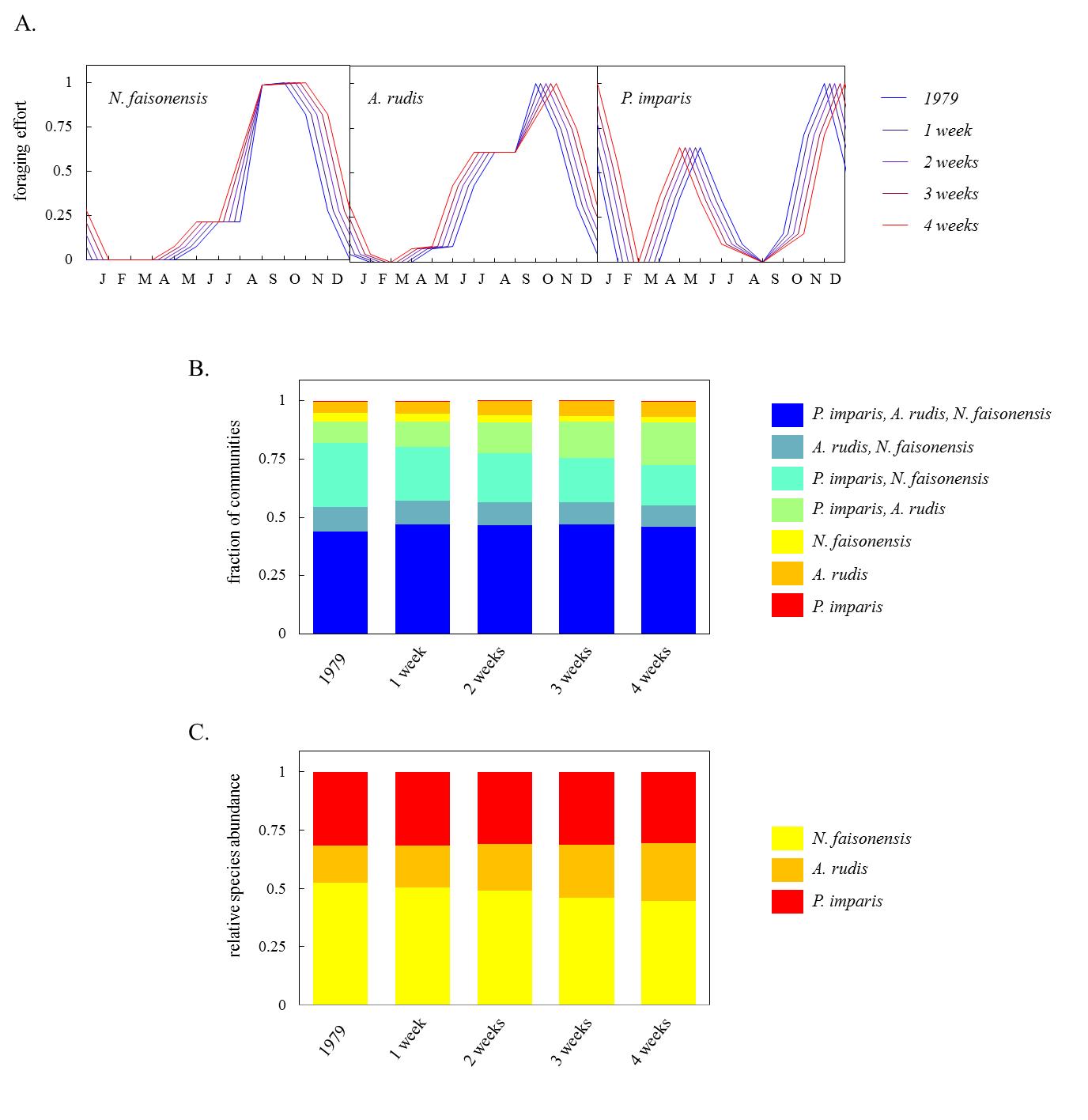


**Figure A.2** (A) Seasonal foraging activities of the three ant species from SERC under spring and fall phenological shifts of 1-4 weeks (i.e. extended summers of 2-8 weeks). (B) Fraction of microhabitats with each possible species combination for each different phenological shift (C) Abundance of each species averaged across all microhabitats for each different phenological shift.

**
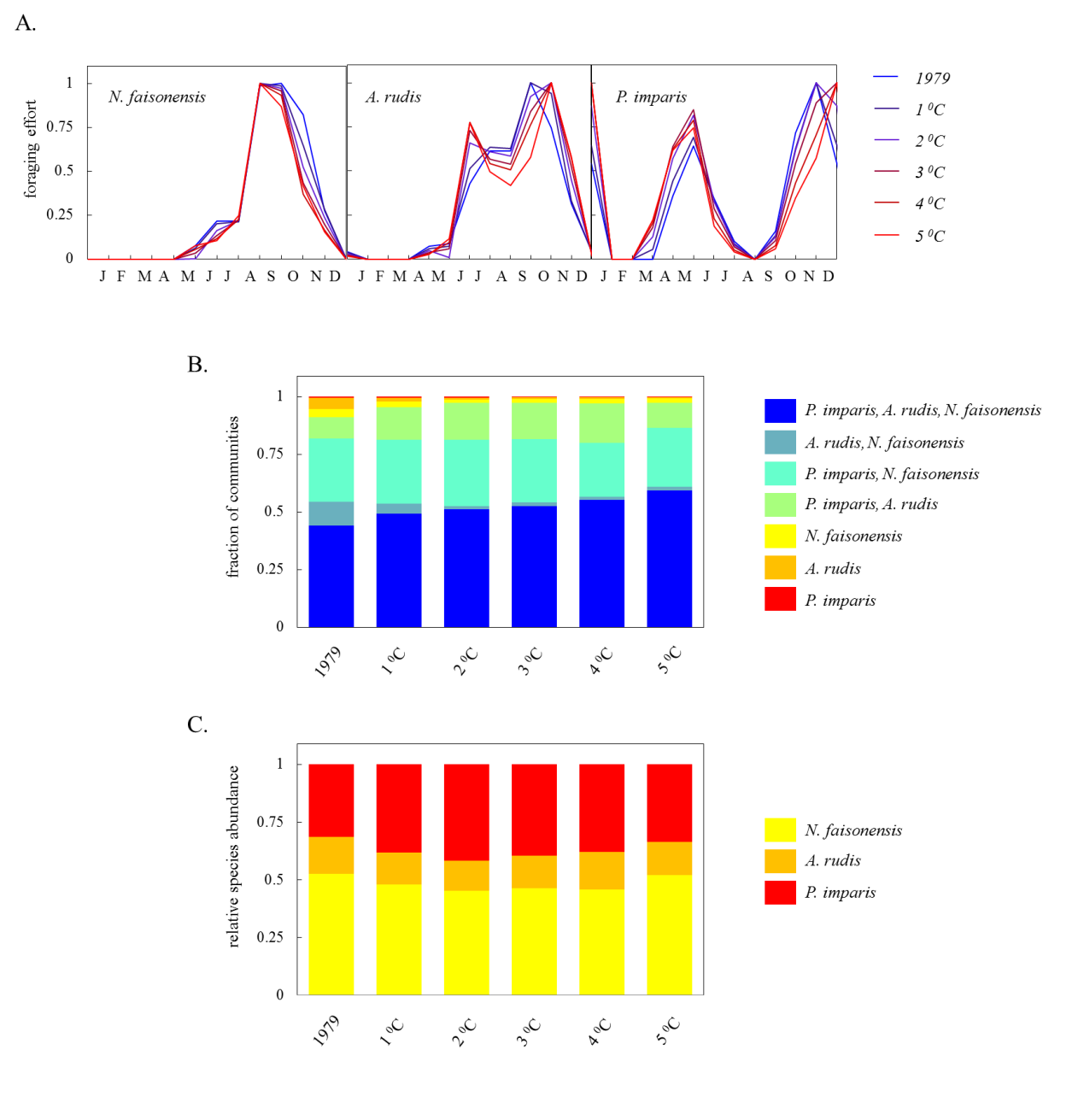
**

**Figure A.3** (A) Seasonal foraging activities of the three ant species from SERC under year-wide temperature increases of 1 – 5°C. (B) Fraction of microhabitats with each possible species combination for each different temperature increase (C) Abundance of each species averaged across all microhabitats for each different temperature increase.
