# Supplementary figures and images for: Predicting future coexistence in a North American ant community
Source: Ecol Evol. 2014 Apr 16;4(10):1804–19. doi: 10.1002/ece3.1048 (PMC4063477; doi:10.1002/ece3.1048)

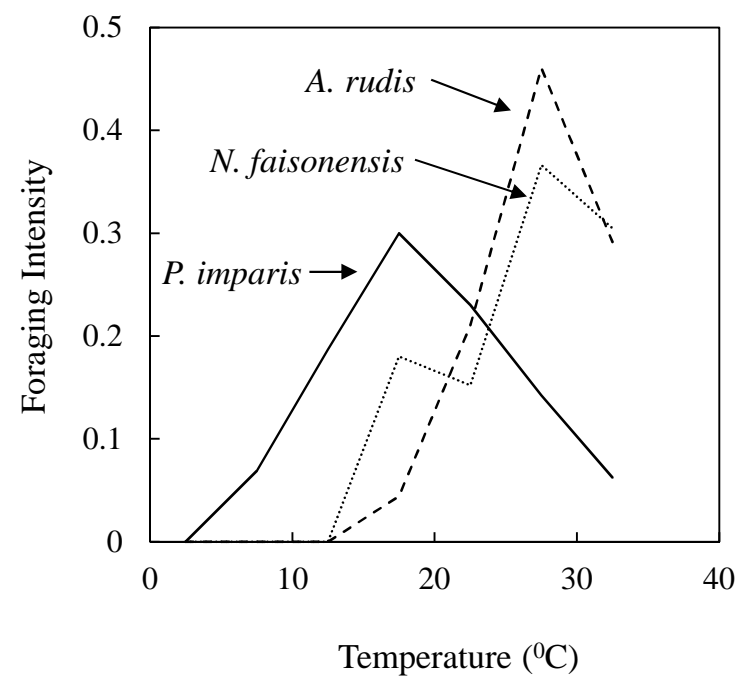

Supplement: Supplementary file 2 [file ece30004-1804-SD2.pdf]

A.

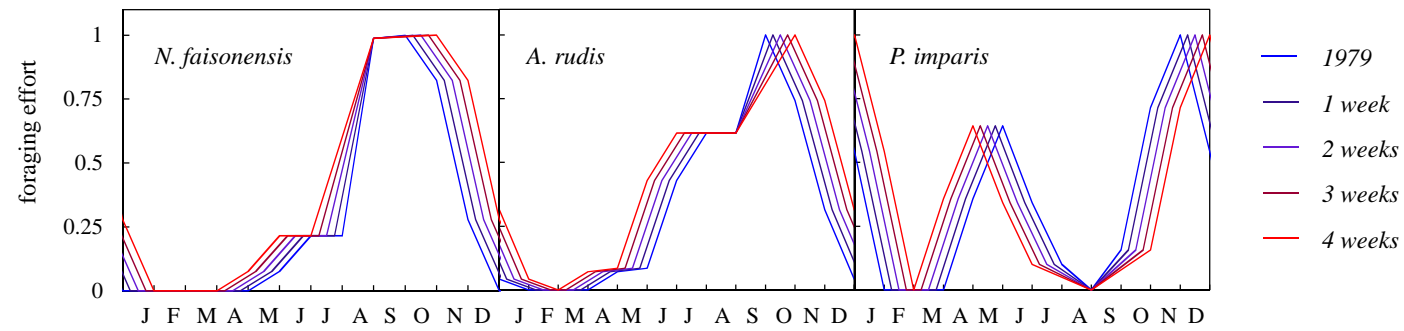

B.

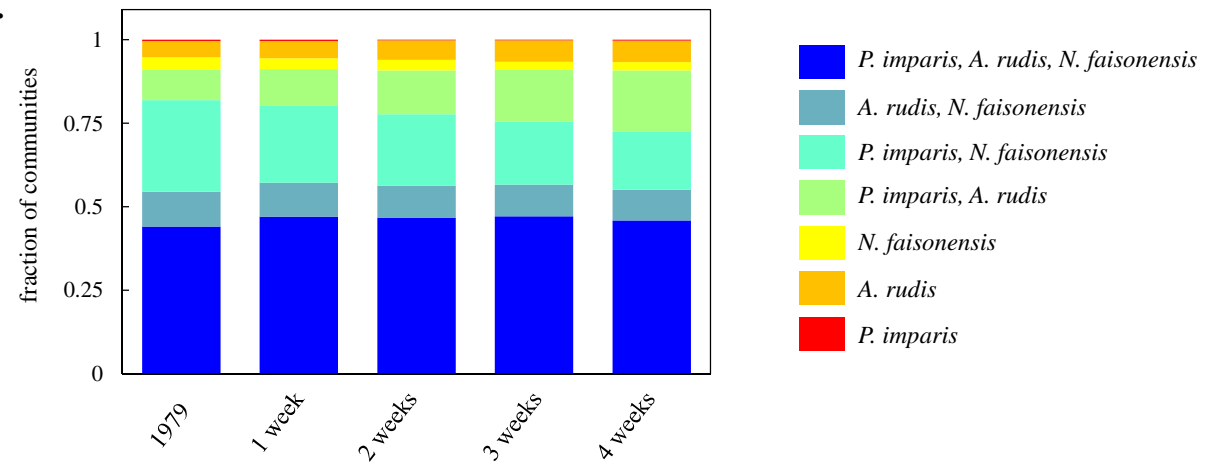

C.

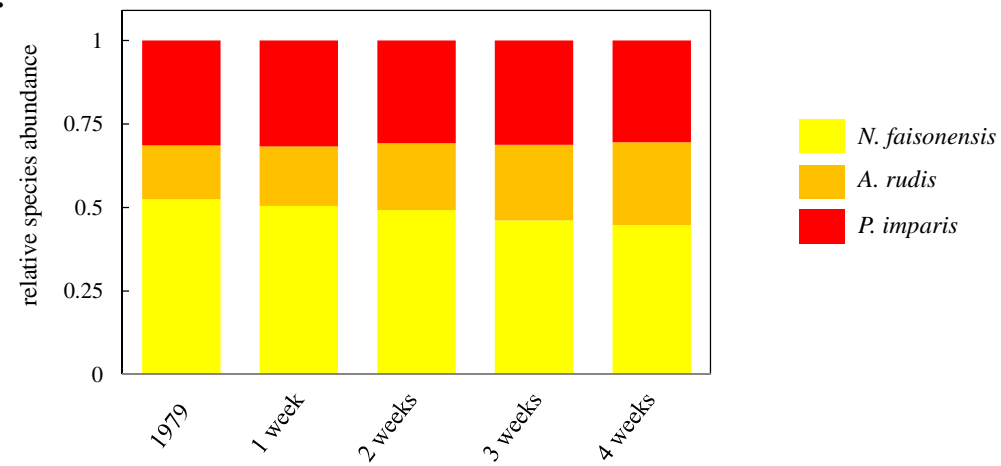

Supplement: Supplementary file 3 [file ece30004-1804-SD3.pdf]

A.

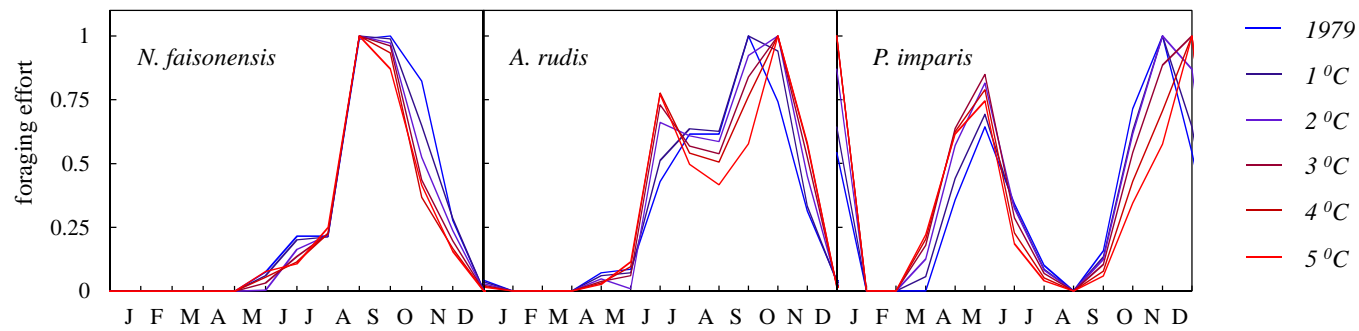

B.

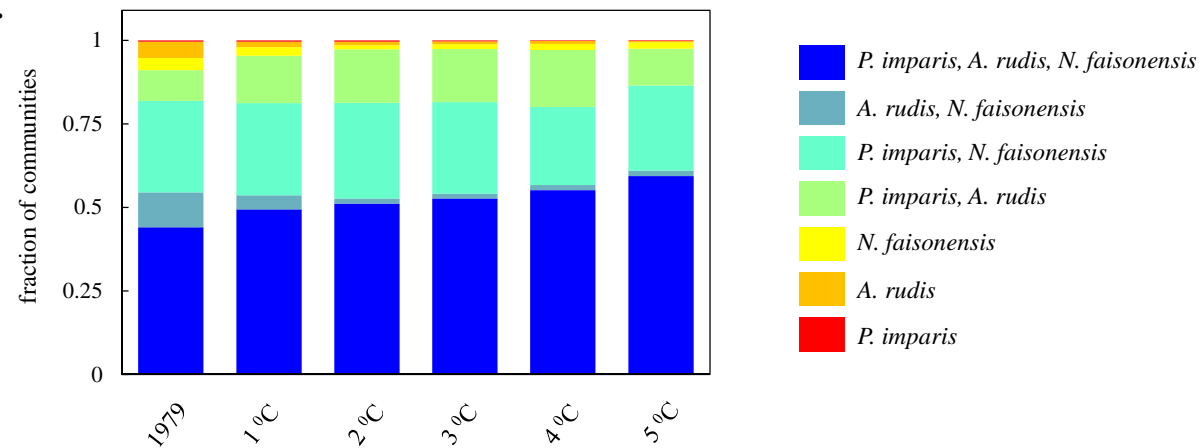

C.

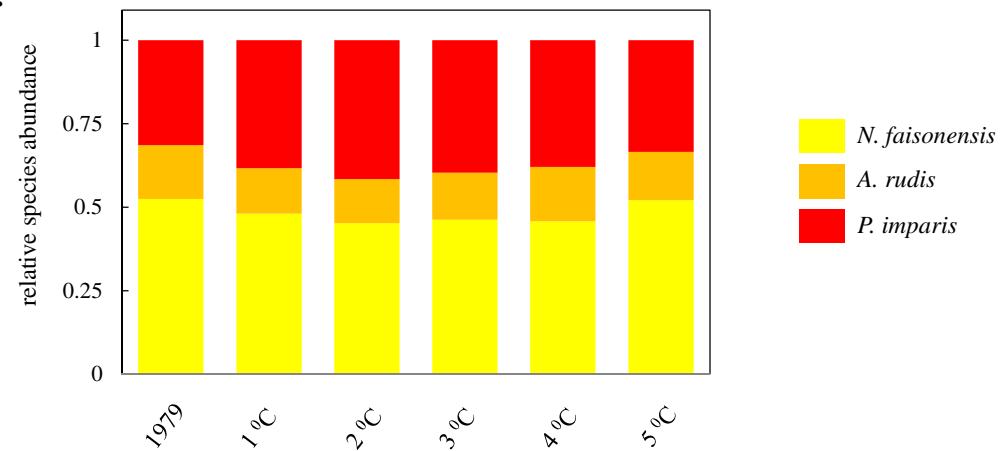

Supplement: Supplementary file 4 [file ece30004-1804-SD4.pdf]
